# Accuracy of parent-reported information for estimating prevalence of overweight and obesity in a race-ethnically diverse pediatric clinic population aged 3 to 12

**DOI:** 10.1186/s12887-015-0320-0

**Published:** 2015-02-12

**Authors:** Nancy P Gordon, R Grant Mellor

**Affiliations:** Division of Research, Kaiser Permanente Medical Care Program, 2000 Broadway, Oakland, CA 94611 USA; Department of Pediatrics, Kaiser Permanente Northern California Central Valley Area, Stockton, CA USA

## Abstract

**Background:**

There is conflicting evidence about the accuracy of estimates of childhood obesity based on parent-reported data. We assessed accuracy of child height, weight, and overweight/obesity classification in a pediatric clinic population based on parent data to learn whether accuracy differs by child age and race/ethnicity.

**Methods:**

Parents of patients ages 3–12 (n = 1,119) completed a waiting room questionnaire that asked about their child’s height and weight. Child’s height and weight was then measured and entered into the electronic health record (EHR) by clinic staff. The child’s EHR and questionnaire data were subsequently linked. Accuracy of parent-reported height, weight, overweight/obesity classification, and parent perception of child’s weight status were assessed using EHR data as the gold standard. Statistics were calculated for the full sample, two age groups (3–5, 6–12), and four racial/ethnic groups (nonHispanic White, Black, Latino, Asian).

**Results:**

A parent-reported height was available for 59.1% of the children, weight for 75.6%, and weight classification for 53.0%. Data availability differed by race/ethnicity but not age group. Parent-reported height was accurate for 49.2% of children and weight for 58.2%. Latino children were less likely than nonHispanic Whites to have accurate height and weight data, and weight data were less accurate for 6–12 year than 3–5 year olds. Concordance of parent- and EHR-based classifications of the child as overweight/obese and obese was approximately 80% for all subgroups, with kappa statistics indicating moderate agreement. Parent-reported data significantly overestimated prevalence of overweight/obesity (50.2% vs. 35.2%) and obesity (32.1% vs. 19.4%) in the full sample and across all age and racial/ethnic subgroups. However, the percentages of parents who perceived their child to be overweight or very overweight greatly underestimated actual prevalence of overweight/obesity and obesity. Missing data did not bias parent-based overweight/obesity estimates and was not associated with child’s EHR weight classification or parental perception of child’s weight.

**Conclusions:**

While the majority of parents of overweight or obese children tend to be unaware that their child is overweight, use of parent-reported height and weight data for young children and pre-teens will likely result in overestimates of prevalence of youth overweight and obesity.

**Electronic supplementary material:**

The online version of this article (doi:10.1186/s12887-015-0320-0) contains supplementary material, which is available to authorized users.

## Background

Childhood obesity is a risk factor for childhood and adult chronic diseases [1,2]. According to population health surveys, the youth obesity rate in the United States has nearly tripled over the last two decades, although it has recently shown signs of leveling off [3]. For cost and logistical reasons, national and state population health surveillance tools that are used to monitor, research, and formulate policy regarding childhood overweight and obesity rely on parent report of a child’s height and weight to create estimates of obesity prevalence. However, there has been little assessment of the accuracy of these statistics for the 3- to 12-year-old age span that reflects the population for most pediatric clinic and school-based obesity intervention efforts. The accuracy of parent-reported height and weight data also has implications for non-surveillance purposes, e.g., identification of children at risk for obesity and obesity-related chronic conditions based on health assessment questionnaires and pediatric obesity-related research and program evaluation.

Published studies of the accuracy of proxy reports of child height, weight, and obesity status have shown that parent-reported values for classifying children as overweight or obese have relatively poor accuracy, including both overestimates and underestimates of overweight and obesity [4-15]. Most of those studies focused on accuracy of weight classification and did not provide information about the accuracy of parent-reported height and weight as separate outcomes, and most did not examine multi-ethnic populations. Given current policy concerns about childhood overweight and obesity, it is important to learn about the accuracy of parent-based information and to learn whether parents have tools at home that can be used to provide more accurate measurements of child height and weight for surveys and research studies upon request.

To assess the accuracy of parent-reported information about child’s height and weight and overweight/obesity classification based on that information, in 2013 we conducted a waiting-room survey with a convenience sample of parents of children ages 3 to 12 in an outpatient clinic of a Northern California health plan. The children were going to have their height and weight measured that day as a routine part of their pediatric visit. The study assessed: (1) Accuracy of parent-reported child height, weight, and resulting overweight/obese and obese classifications in children ages 3 to 12, with clinic-measured height, weight, and overweight/obese and obese classifications as the standard; (2) Factors associated with accuracy of parent-reported data; (3) Accuracy of parents’ perceptions about whether their children are overweight; (4) Factors associated with missing weight classification data and the extent to which missing weight classification data introduces bias into overweight/obesity estimates; (5) Availability of tools at home (scale, tape measure) to measure a child’s height and weight if asked to do so; and (6) Whether assessments 1–5 differ by children’s age group and race/ethnicity.

## Methods

### Data source

This study was implemented in three Kaiser Permanente Northern California pediatric clinics (Stockton, Vallejo, and Fairfield) that serve a race-ethnically diverse population that is primarily working and middle class. All three clinics routinely measure height and weight at every pediatric appointment. From January to April 2013, pediatric department receptionists and medical assistants handed out a brief (13 item) paper questionnaire in English or Spanish to all parents of pediatric patients ages 3 to 12 at time of registration for the visit. The receptionists asked parents of age-eligible patients if they would be willing to fill out a very short questionnaire about their child’s height and weight while they were in the waiting room and mentioned a small thank-you gift they would receive. If the parent agreed, the receptionist put the pediatric patient’s name, health plan number, and appointment date on the questionnaire and handed it to the parent on a clipboard. Parents were told to return their completed questionnaire to the receptionist or medical assistant before their child was weighed and measured, at which time they would receive the gift.

Parents were informed at the top of the questionnaire that the study was being done to learn how accurate parents are when they are asked to report their child’s height and weight in surveys and to medical staff during phone consults, and that their answers would be linked to their child’s height and weight measured by the medical assistant. The questionnaire (see Additional file 1) asked for the child’s age, sex, height (in feet/inches or meters/centimeters), weight (in pounds/ounces or kilograms/grams), the last time the parent found out the child’s height and weight, parent’s perception of the child’s weight (underweight, about right, overweight, or very overweight), and whether they had a scale and a tape measure or yard stick at home that could be used to weigh and measure the child if they were asked to do so.

At each site, point-of-service staff were trained in the data collection procedures and monitored by the Pediatric department manager or Pediatric Chief. Medical assistants were instructed not to give parents who were participating in the study access to the child’s clinic-measured height and weight until after the questionnaire was collected. Data collection lasted 2 to 3 consecutive weeks at each site, at which time completed questionnaires were sent to the Study Director. Questionnaire data were subsequently linked with height, weight, and body mass index (BMI) measurements from the child’s electronic health record (EHR) at that visit, as well as age, sex, and race/ethnicity. The study protocol was approved by Kaiser Permanente Northern California Region’s Institutional Review Board.

### Statistical analysis

BMI-for-age percentiles based on child’s height, weight, age, and gender were calculated from parent-reported and EHR data by using SAS code available from the Centers for Disease Control (CDC) [16]. Individual differences between parent-reported and EHR values for height, weight, and BMI-for-age percentile were calculated by subtracting the EHR value from the parent-reported value. Parent-reported data was considered to be accurate when reported height was within ±1 inch of the EHR height, weight was within ±2 lbs. of the EHR weight, and BMI-for-age percentile was within ±5 percentage points of EHR BMI-for-age percentile. Based on their BMI-for-age percentile, children were classified as not overweight (1st–84th percentile), overweight/obese (≥85th percentile), or obese (≥95th percentile). Children with a BMI-for-age percentile <1, which usually results from a biologically implausible height, weight, or height-weight combination, were not assigned a weight classification following CDC recommendations [16]. Accuracy of overweight/obese and obese classification based on parent-reported and EHR data was assessed using kappa statistics [17]. Using EHR weight classification as a “gold standard”, we calculated sensitivity (probability that a child who is overweight/obese or obese is accurately classified as such based on parent-reported data), specificity (probability that child who is not overweight/obese or obese is accurately classified as such based on parent-reported data), and positive predictive value (probability that a child who is classified as overweight/obese or obese based on parent-reported data was accurately classified). We compared prevalence of overweight/obesity and obesity based on EHR data (all children and children with a parent report-based weight classification), on parent-reported data (for children with a usable BMI-for-age percentile), and parent perception of whether the child was overweight in the full sample, two age groups (3 to 5 years and 6 to 12 years), and four racial and ethnic groups (nonHispanic White, Black, Latino, and Asian).

Because 32% to 56% of children in different age and race-ethnic subgroups had insufficient parent-reported data to assign a weight classification, we also assessed whether missing parent-reported data biased prevalence estimates of overweight/obesity and obesity for the full sample and different demographic subgroups. To do this, we compared the EHR-based prevalence of overweight/obesity and obesity for groups of children who did and did not have a usable weight classification based on parent-reported data. We also re-estimated parent report-based prevalence of overweight/obesity and obesity using a post-stratification weighting factor that made the sample of children with parent report-based weight classification reflect the actual age group (3 to 5, 6 to 9, 10 to 12), sex, and racial/ethnic distribution of the full sample [18]. Finally, to examine factors associated with missing weight classification data, we compared children with and without parent report-based weight classification on parent perception that their child was overweight; length of time since child’s most recent height and weight measurements; parent who completed the questionnaire; and where relevant, child’s sex, age group, and race/ethnicity.

An online statistics program [19] was used to calculate kappa, sensitivity, specificity, positive predictive value using data from 2 × 2 tables. All other statistical analyses were performed using SAS version 9.3 [20]. Chi-square tests were used to assess whether differences between age groups (3 to 5 vs. 6 to 12) and between nonHispanic Whites and each of the other race/ethnic groups on categorical variables were statistically significant. Two-tailed z-tests for proportions were used to test for differences between prevalence of overweight/obesity and obesity based on EHR data for the full sample and parent-reported data, and two-tailed t-tests were used to compare means and mean differences. Multivariable logistic regression and general linear models were used to assess independent association of demographic and other factors with accuracy of parent-reported data. Unless otherwise specified, differences cited in the text as statistically significant met the *P* < .05 threshold. We did not adjust *P*-values for multiple comparisons, but the results of all planned race-ethnicity and age group comparisons are reported in the tables or text.

## Results

### Study sample characteristics

Questionnaires were collected for 1,119 children aged 3 to 12. However, 67 (6%) of these were later excluded due to the questionnaire having been completed by a non-parent/guardian (n = 39), too much missing information (n = 13), medical record number that couldn’t be matched to an appointment (n = 1), no height in the child’s EHR for the date on the questionnaire (n = 3), or implausible (<1st) EHR-derived BMI-for-age percentile (n = 11). This left information for 1,053 children, 434 aged 3 to 5 (210 boys, 224 girls) and 619 aged 6 to 12 (313 boys, 306 girls). Of the 1,021 children (97%) who could be matched to a race/ethnicity, 27.0% were nonHispanic White (n = 276), 11.4% African-American/Black (n = 116), 40.1% Hispanic/Latino (n = 409), 19.3% Asian (n = 197), and 5.2% Other (n = 55). The racial/ethnic composition of the two age groups and the age-gender group distribution within the race-ethnic groups were not significantly different. Most (84.6%) of the questionnaires were completed by a mother, with the rest completed by a father (15.3%) or other guardian (0.1%).

### Completeness of parent-reported data

Of the 1,053 children with complete EHR data, 59.1% had a parent-reported height, 75.6% had a parent-reported weight, 56.3% had both height and weight, and 21.6% had neither (Table 1). Only 53% of children had usable BMI-for-age percentile for assignment to a weight classification after 35 (3%) of children were excluded due to having a value below the 1st percentile.

**Table 1:** **Availability of parent-reported child height and weight data, when parents recall last obtaining these measures, and source of parent report**

		**By child age**		**By child race/ethnicity**			
	**All**	**Ages 3–5 y**	**Ages 6–12 y**	**NonHispanic White**	**Black**	**Latino**	**Asian**
	**(N = 1053)**	**(N = 434)**	**(N = 619)**	**(N = 276)**	**(N = 116)**	**(N = 409)**	**(n = 197)**
	**% (n)**	**% (n)**	**% (n)**	**% (n)**	**% (n)**	**% (n)**	**% (n)**
Height	59.1 (622)	59.0 (256)	59.1 (366)	71.4 (197)	64.7 (75)	52.8^b^ (216)	51.3^b^ (101)
Weight	75.6 (796)	79.5 (345)	73.2^a^ (453)	88.0 (243)	75.4^b^ (68)	70.2^b^ (287)	70.6^b^ (139)
Usable BMI-for-age percentile data for weight classification^1^	53.0 (558)	51.8^a^ (225)	53.8 (333)	68.5 (189)	58.6 (68)	45.5^b^ (186)	43.1^b^ (85)
Perception of whether child is overweight	98.1 (1033)	99.5 (432)	97.1 (601)	99.6 (275)	100.0 (116)	96.1 (393)^b^	98.5 (194)
Last learned child’s weight							
Within past 7 days	20.0 (211)	22.8 (99)	18.1 (112)	22.5 (62)	16.4 (19)	15.7^b^ (64)	27.9 (55)
>7 days but within past month	27.5 (289)	28.6 (124)	26.7 (165)	26.4 (73)	25.0 (29)	25.7 (105)	32.5 (64)
>1 month but within past 6 months	31.0 (325)	32.5 (141)	29.9 (185)	35.9 (99)	36.2 (42)	31.5 (129)	19.8 (39)
More than 6 months ago	18.9 (199)	14.1 (61)	22.3^a^ (138)	13.4 (37)	19.8 (23)	23.2^b^ (95)	18.3 (36)
Not reported	2.6 (28)	2.0 (9)	3.0 (19)	1.8 (5)	2.6 (3)	3.9 (16)	1.5 (3)
Last learned child’s height							
Within past 7 days	13.9 (146)	15.4 (67)	12.8 (79)	14.5 (40)	12.9 (15)	12.0 (49)	18.3 (36)
>7 days but within past month	20.1 (212)	21.4 (93)	19.2 (119)	21.4 (59)	18.1 (21)	18.6 (78)	23.4 (46)
>1 month but within past 6 months	33.3 (348)	36.9 (60)	30.4 (188)	40.9 (113)	37.9 (44)	31.3 (128)	20.8 (41)
More than 6 months ago	28.4 (299)	22.6 (98)	32.5^a^ (201)	19.9 (55)	28.4 (33)	32.5^b^ (133)	32.0^b^ (63)
Not reported	4.5 (48)	3.7 (16)	5.1 (32)	3.3 (9)	2.6 (3)	5.6 (23)	5.6 (11)
Mother reporting	84.6 (891)	83.9 (364)	85.1 (527)	80.4 (222)	88.8^b^ (103)	89.7^b^ (367)	78.2 (154)

^1^BMI-for-age percentiles <1 were considered biologically implausible values and excluded from weight classification analyses.

^a^Significantly different from 3 to 5 year olds by chi-square test (*P* < .05).

^b^Significantly different from nonHispanic Whites by chi-square test (*P* < .05).

Availability of parent-reported height and weight data and usable BMI-for-age percentile did not significantly differ by child age group. However, compared to parents of nonHispanic White children, parents of Black, Latino and Asian children were significantly less likely to report their child’s weight, and parents of Latino and Asian children were significantly less likely to report a height. This resulted in significantly lower percentages of Latino (45%) and Asian (43%) children compared to nonHispanic White children (68%) for whom a BMI-for-age percentile could be calculated and weight classification assigned based on parent-reported data.

As shown in Table 1, approximately 47% of parents indicated they last learned their child’s weight within the past month, but for nearly 20% of parents it had been over 6 months. Similarly, 34% had learned their child’s height within the past month, but for nearly 30% it had been over 6 months. Parents of children aged 6 to 12 were significantly more likely than parents of children aged 3 to 5 to indicate that these measurements had last occurred more than six months ago. As the length of time since last known measurements increased (in past 7 days, >7 days but within past month, >1 month but within past 6 months, more than 6 months ago), there were statistically significant declines in the percentages of parents who reported child weight (93.8%, 86.2%, 73.3%, and 43.7%, respectively), height (84.2%, 70.9%, 62.6%, and 37.1%, respectively), and sufficient information to categorize the child as overweight/obese or obese (79.5%, 63.7%, 54.6%, and 33.1%, respectively). About 18% of parents said their child had grown a lot taller since his/her height was last measured, with no significant difference by child age or gender (data not shown).

### Accuracy of parent-reported information for height, weight, and obesity classification

Measures of the accuracy of parent-reported height and weight and calculated BMI-for-age percentile and classification as overweight or obese are shown in Table 2. Parent-reported child height was within 1 inch of EHR height for 49% of children and parent-reported child weight was within 2 lbs. of the EHR weight for slightly under 60% of children. Errors for both height and weight were more often due to underestimation than overestimation. Approximately 35% of parents (220/662) underestimated actual height by at least 1 inch and 26% by at least 2 inches (mean height difference of -1.10, SD = 3.70), with no significant difference by age group. About 22% (74/343) of parents of children aged 3 to 5 and 39% (175/452) of parents aged 6 to 12 underestimated their child’s weight by at least 2 lbs., with mean weight difference significantly smaller for the younger versus older children (-0.73 (SD 3.14) vs. -2.06 (SD 6.75), *P* < .0001). BMI-for-age percentile based on parent report was within ±5 percentiles for approximately 46% (259/558) of children, but accuracy was significantly higher for the older versus younger children (53.8% vs. 35.5%, *P* < .0001) and for Black versus nonHispanic White children (55.9% vs. 40.8%, *P* < .05). Most errors were due to parent-based BMI-for-age percentiles >5 percentile points higher than the EHR. For the full sample (n = 558) and across demographic subgroups, children were accurately classified as overweight/obese and obese based on parent-reported data approximately 80% of the time, with misclassification error more often due to children being classified as overweight/obese or obese based on parent-reported data when they were not.

**Table 2:** **Accuracy of parent-reported data for child height, weight, BMI-for-age percentile, and weight classification as compared to electronic health record data**

		**By child age**		**By child race/ethnicity**			
**Accuracy of parent-reported data**	**All**	**Age 3–5 y**	**Age 6–12 y**	**NonHispanic White**	**Black**	**Latino**	**Asian**
	**%**	**%**	**%**	**%**	**%**	**%**	**%**
	**(N = 622)**	**(N = 256)**	**(N = 366)**	**(N = 197)**	**(N = 75)**	**(N = 216)**	**(N = 101)**
Height^1^ Within ±1 inch of EHR height	49.2	46.1	51.4	52.8	50.7	42.6^b^	53.5
Underestimates EHR height by >1 inch	35.4	36.3	34.7	34.0	41.3	37.0	31.7
Overestimates EHR height by >1 inch	17.6	17.6	13.9	13.2	8.0	20.4	14.8
Mean (SD) difference of parent-reported vs. EHR height	-1.1 (3.7)	-1.0 (3.7)	-1.2 (3.7)	-0.8 (2.9)	-2.1^b^ (3.9)	-1.3 (4.4)	-1.0 (3.2)
Weight^1^	**(N = 796)**	**(N = 343)**	**(N = 452)**	**(N = 241)**	**(N = 87)**	**(N = 287)**	**(N = 139)**
Within ± 2 lbs. of EHR weight	58.2	68.2	50.7^a^	60.6	59.8	51.6^b^	62.6
Underestimates EHR weight by > 2 lbs.	31.3	21.6	38.6^a^	28.6	31.0	37.9^b^	25.9
Overestimates EHR weight by > 2 lbs.	10.4	10.2	10.8	10.8	9.2	10.2	11.5
Mean (SD) difference of parent-reported vs. EHR weight	-1.5 (6.4)	-0.7 (3.1)	−2.1^a^ (8.1)	-0.9 (7.5)	-2.0 (5.1)	-2.2^b^ (5.6)	-1.1 (5.4)
BMI-for-age percentile^1^	**(N = 558)**	**(N = 225)**	**(N = 333)**	**(N = 189)**	**(N = 68)**	**(N = 186)**	**(N = 85)**
Within ± 5 percentile pts. of EHR	46.4	35.5	53.8^d^	40.8	55.9^b^	47.3	47.1
Underestimates EHR by > 5 percentiles	19.0	19.6	18.6	25.9	8.8^b^	17.2^b^	17.6
Overestimates EHR by > 5 percentiles	34.4	44.9	27.6^d^	33.3	35.3	35.5	35.3
Mean (SD) difference of parent-report vs. EHR-based BMI-for-age percentile	5.5 (26.5)	9.7 (30.4)	2.7^a^ (23.2)	3.1 (25.2)	10.7^b^ (27.3)	5.1 (26.6)	7.1 (29.4)
Weight classification^1^	**(N = 558)**	**(N = 225)**	**(N = 333)**	**(N = 189)**	**(N = 68)**	**(N = 186)**	**(N = 85)**
Overweight/obese classification matched EHR^2b^	79.0	75.6	81.4	79.4	80.9	76.9	80.0
Child misclassified as not overweight/obese	3.8	2.2	4.8	4.2	1.5	4.8	3.5
Child misclassified as overweight/obese	17.2	22.2	13.8^a^	16.4	17.6	18.3	16.5
Obesity classification matched EHR^2b^	81.9	80.0	83.2	87.3	80.9	78.5^b^	78.8
Child misclassified as not obese	3.4	2.2	4.2	3.2	4.4	3.2	2.4
Child misclassified as obese	14.7	17.8	12.6	9.5	14.7	18.3^b^	18.8^b^

EHR = Electronic health record; SD = Standard deviation around mean difference.

^1^Restricted to children with valid parent-reported data.

^2^Children with a BMI-for-age percentile ≥ 85 were classified as overweight/obese and ≥ 95 as obese.

^a^Significant difference between age groups by chi-square test (*P* < .05).

^b^Significantly different from nonHispanic Whites by chi-square test (*P* < .05).

Kappa statistics showed only moderate levels of agreement for overweight/obese (range 0.50–0.63) and obese (range 0.44–0.60) classifications based on parent-reported and EHR data for the full sample and most subgroups (Table 3). For the overweight/obese classification, sensitivity ranged from the high 80s to mid-90s, specificity from the high 60s to mid-70s, and positive predictive values from the low 50s to mid-70s. For the obesity classification, sensitivity ranged from the mid-70s to mid-80s, specificity from the mid-70s to high 80s, and positive predictive values from the low 40s to low 60s. Positive predictive values for overweight/obese and obesity classifications were significantly higher for the 6 to 12 year olds than the 3 to 5 year olds.

**Table 3:** **Accuracy and validity of child overweight and obesity classification based on parent-reported data as compared to electronic health record data**

		**By child Age**		**By child race/ethnicity**			
**Weight classification**	**All**	**Ages 3–5 y**	**Ages 6–12 y**	**NonHispanic White**	**Black**	**Latino**	**Asian**
	**(N = 558)**	**(N = 225)**	**(N = 333)**	**(N = 189)**	**(N = 68)**	**(N = 186)**	**(N = 85)**
	**% (95% CI)**	**% (95% CI)**	**% (95% CI)**	**% (95% CI)**	**% (95% CI)**	**% (95% CI)**	**% (95% CI)**
Overweight/obese (BMI-for-age percentile ≥ 85)							
Kappa	0.58 (0.53–0.63)	0.50 (0.39–0.55)	0.63 (0.54–0.70)	0.57 (0.44–0.66)	0.63 (0.41–0.68)	0.55 (0.42–0.64)	0.60 (0.39–0.70)
Sensitivity	89.8 (85.3–93.2)	91.7 (82.1–96.8)	89.0 (83.7–93.0)	86.7 (76.8–93.3)	96.4 (82.9–99.8)	88.9 (81.3–94.2)	90.0 (75.6–97.3)
Specificity	72.8 (70.2–74.8)	69.7 (66.2–71.6)	75.5 (71.5–78.6)	76.0 (71.4–79.1)	70.0 (60.5–72.4)	67.6 (61.8–71.7)	74.5 (66.7–78.5)
Positive predictive value	65.7 (62.5–68.2)	52.4 (46.9–55.3)	73.7^a^ (69.4–77.0)	62.7 (55.5–67.5)	69.2 (59.5–71.7)	67.9 (62.2–71.9)	65.9 (55.3–71.2)
Obese (BMI-for-age percentile ≥ 95)							
Kappa	0.54 (0.47–0.60)	0.44 (0.31–0.51)	0.60 (0.50–0.68)	0.56 (0.39–0.68)	0.55 (0.30–0.69)	0.53 (0.40–0.61)	0.48 (0.26–0.58)
Sensitivity	83.6 (76.4–89.3)	84.4 (68.1–94.0)	83.3 (74.9–89.7)	78.6 (61.1–90.4)	84.2 (63.4–95.6)	87.8 (76.4–94.7)	87.5 (63.4–97.8)
Specificity	81.4^c^ (79.5–82.9)	79.3^c^ (76.6–80.9)	83.1^c^ (80.3–85.3)	88.3^c^ (85.3–90.3)	78.0^b^ (70.1–82.3)	75.2^b^ (71.1–77.7)	76.8 (71.2–79.2)
Positive predictive value	54.2^c^ (49.5–57.8)	40.3^c^ (32.5–44.9)	62.5^ac^(56.2–67.3)	53.7 (41.7–61.8)	59.3 (44.6–67.3)	55.8^c^ (48.6–60.3)	46.7^c^ (33.8–52.1)

Notes: All analyses restricted to children with data from both sources; CI = Confidence interval; Kappa statistic is not a percentage.

^a^Significant difference between age groups by *t*-test (*P* < .05).

^b^Significantly different from nonHispanic Whites by *t*-test (*P* < .05).

^c^Significantly different from same statistic for Overweight/obese by *t*-test (*P* < .05).

Multivariable logistic regression models that included the child’s race/ethnicity, age group, and sex were used to assess statistical significance of demographic differences in accuracy of height, weight, and overweight/obese and obese classifications. Children aged 6 to 12 were significantly less likely than 3 to 5 year olds to have an accurately reported weight (OR = 0.47, CI: 0.35–0.63), but did not significantly differ from the younger children with regard to accuracy of height or overweight/obese and obese classifications. Boys were significantly less likely than girls to have an accurately reported height (OR = 0.63, CI: 0.46–0.87) and obese classification (OR = 0.54, CI: 0.35–0.84), but did not significantly differ in accuracy of reported weight or overweight/obese classification. Compared to nonHispanic white children, Latino children were significantly less likely to have an accurately reported height (OR = 0.67, CI: 0.45–0.99), weight (OR = 0.69, CI: 0.46–0.98), and obese classification (OR = 0.55, CI: 0.31–0.95), but did not significantly differ on overweight/obese classification. Accuracy of parent-reported height and weight data and overweight/obese and obese classification for Black and Asian children was not significantly different from nonHispanic Whites. The strength of association of these demographic factors with accuracy was not mediated by parent sex (same or opposite child sex) or recentness of the parent learning their child’s height/weight. However, accuracy of parent reported weight and height was significantly lower when parents last learned their child’s measurements > 7 days vs. ≤ 7 days before the survey (OR = 0.30, CI: 0.21–0.44 for weight and OR = 0.26, CI: 0.17–0.41 for height).

### Prevalence of overweight/obesity and obesity based on EHR and parent-reported data

For the full sample and most subgroup comparisons, there were significant differences (15 percentage points on average) in prevalence of overweight/obesity and obesity based on parent-reported data for those children with usable BMI-for-age percentile data versus EHR data for all children in the sample (Table 4). Based on EHR data for the full sample, 35.2% of the children were classified as overweight/obese, with 19.4% in the obese range, compared with significantly higher prevalence of 50.2% and 32.1%, respectively, using parent-reported data. EHR data indicated that children aged 6 to 12 were significantly more likely than 3 to 5 year olds to be overweight/obese (41% vs. 27%, *P* < .0001) and obese (23.6% vs. 13.4%, *P* < .0001), but prevalence differences by age group were not as large or statistically significant when parent-reported data were used (52.5% vs. 46.7% overweight/obese, 33.6% vs. 29.8% obese). Comparisons of overweight/obesity and obesity across race-ethnic groups generally showed smaller differences in point prevalence between EHR data and parent-reported data, in some instances resulting in race-ethnic differences being statistically significant only using parent-reported data. For example, Latinos were significantly more likely to be overweight/obese and obese than nonHispanic Whites based on both data sources, but differences between nonHispanic Whites and Blacks were significant for obesity based only on parent-reported data.

**Table 4:** **Prevalence of child overweight and obesity based on electronic health record and parent-reported data**

		**By child age**		**By child race/ethnicity**			
	**All**	**Ages 3–5 y**	**Ages 6–12 y**	**NonHispanic White**	**Black**	**Latino**	**Asian**
	**% (95% CI)**	**% (95% CI)**	**% (95% CI)**	**% (95% CI)**	**% (95% CI)**	**% (95% CI)**	**% (95% CI)**
Overweight/obese (BMI- for-age percentile ≥ 85)							
EHR data for full sample	35.2 (32.3–38.1)	27.0 (22.8–31.1)	41.0^a^ (37.1–44.9)	29.7 (24.3–35.1)	31.9 (23.3–40.5)	42.8^b^ (38.0–47.6)	32.0 (25.4–38.5)
Parent-reported data	50.2^c^ (46.0–54.3)	46.7^c^ (40.1–53.2)	52.5^a^ (47.2–57.9)	43.9^c^ (36.8–51.1)	57.4^c^ (45.3–69.4)	57.0^cb^ (49.8–64.2)	48.2 (37.4–59.1)
Obese (BMI-for-age percentile ≥ 95)							
EHR data for full sample	19.4 (17.0–21.8)	13.4 (10.1–16.6)	23.6^a^ (20.2–26.9)	14.4 (10.3–18.7)	18.1 (11.0–25.2)	23.7^b^ (19.6–27.9)	19.3 (13.8–24.8)
Parent-reported data	32.1^c^ (28.2–36.0)	29.8^c^ (23.7–35.8)	33.6^c^ (28.5–38.7)	21.6^c^ (15.7–27.5)	39.1^bc^ (27.3–50.9)	41.4b^bc^(34.2–48.5)	35.3^bc^ (25.1–45.5)

EHR = Electronic health record; CI = Confidence interval around percentage.

Denominators for full sample/children with parent-reported weight classification: All: N = 1053/558; Ages 3 to 5: N = 434/225; Ages 6 to 12: N = 619/333.

NonHispanic Whites: N = 276/180; Blacks: N = 116/69; Latinos N = 409/186; Asians N = 197/85.

^a^Significant difference between age groups by two-tailed z-test (*P* < .05).

^b^Significantly different from nonHispanic Whites by two-tailed z-test (*P* < .05).

^c^Significant difference between prevalence estimated from parent-reported data and EHR data for all children in the demographic group by two-tailed z-test (*P* < .05).

### Parent perception of child being overweight

**Figure 1:**
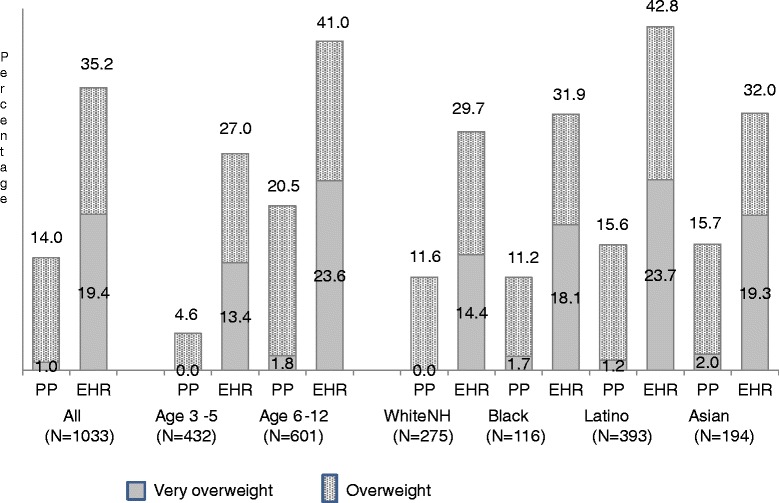
**Comparison of Parent Perception of Child Weight with EHR Weight Classification Percentages of children regarded by their parents or reported in the electronic health record as overweight or very overweight are shown for the indicated categories.** PP, parent perception. EHR, electronic health record. WhiteNH, nonHispanic White.

Data about parent perception of whether the child was of normal weight, overweight, or very overweight was available for nearly all children. The percentages of children whose parents thought they were overweight (14.0%) or very overweight (1.0%) were significantly lower than the percentages with those weight classifications based on EHR data (Figure 1). This was true across age and race-ethnic groups. Children aged 6 to 12 years were significantly more likely than 3 to 5 year olds to be perceived by parents as overweight (20.5% vs. 4.6%, *P* < .0001), and Latinos and Asians were significantly more likely to be perceived as overweight than nonHispanic Whites (15.6% and 15.7% vs. 11.6%, respectively), with no significant difference by child sex. Only 61.4% (121/197) of children classified as obese (EHR BMI-for-age percentile ≥95) were considered by their parent to be overweight, with children in the older age group significantly more likely (OR = 5.38, CI 2.75–10.52) to be considered overweight than the younger children and no significant difference by child race/ethnicity or sex.

### Effect of missing parent-reported data on estimated prevalence of overweight and obesity

Due to the large number of children for whom a weight classification could not be assigned based on parent-reported data, we re-estimated the prevalence of overweight/obesity and obesity using parent-reported data weighted to reflect the age and gender counts for each race-ethnic group in the full sample. These new prevalence estimates for the full sample and for each demographic group (not shown) were nearly identical to those produced with the unweighted data, suggesting no bias.

We also compared children with and without parent-reported weight classification data on the following factors: whether height, weight, or both measures were unavailable; child weight classification status based on EHR; parent perception that the child is overweight; child sex; child age group; and length of time since child’s weight and height were last measured (Table 5). Black children missing a parent-reported weight classification were significantly less likely than those who had one to be classified based on EHR data as overweight/obese and to have their parent think they are overweight. For other demographic subgroups, EHR-based classification as overweight/obese and parent perception that their child was overweight did not significantly differ between children with and without parent-reported weight classification data. Across all demographic subgroups, with the exception of Black children, parents of children without a parent-reported weight classification were significantly more likely than those with one to indicate that it had been more than 6 months since they last learned their child’s height and weight.

**Table 5:** **Comparison of children with and missing overweight and obesity classifications based on parent-reported data**

			**By child age**				**By child race/ethnicity**							
	**All**		**Ages 3–5 y**		**Ages 6–12 y**		**NonHispanic White**		**Black**		**Latino**		**Asian**	
	**PR**	**No PR**	**PR**	**No PR**	**PR**	**No PR**	**PR**	**No PR**	**PR**	**No PR**	**PR**	**No PR**	**PR**	**No PR**
	**(N = 558)**	**(N = 495)**	**(N = 225)**	**(N = 209)**	**(N = 333)**	**(N = 286)**	**(N = 189)**	**(N = 87)**	**(N = 68)**	**(N = 48)**	**(N = 223)**	**(N = 186)**	**(N = 85)**	**(N = 112)**
	**%**	**%**	**%**	**%**	**%**	**%**	**%**	**%**	**%**	**%**	**%**	**%**	**%**	**%**
EHR weight classification														
Overweight/obese	36.7	33.5	26.7	27.3	43.5	38.1	31.7	25.3	41.2	18.8^a^	43.5	42.2	35.3	29.5
Obese	20.8	17.8	14.2	12.4	25.2	21.7	14.8	13.8	27.9	4.2^a^	26.3	21.5	18.8	19.6
Parent thinks child is overweight	17.2	10.8	4.9	4.3	25.8	15.6^a^	14.8	4.6^a^	16.2	4.2^a^	19.4	13.6	21.4	11.8
Child is a boy	48.9	50.5	47.6	49.3	49.9	51.4	47.6	54.0	36.8	54.2	54.8	45.7	48.2	55.4
Mean age (SD) of children	7.5 (3.1)	6.9^a^ (2.7)	4.3 (0.8)	4.3 (0.9)	9.7 (1.9)	8.8^a^ (1.9)	7.3 (3.1)	7.1 (2.5)	7.9 (3.0)	6.8 (2.8)	7.7 (3.1)	7.2 (2.8)	7.4 (3.1)	6.4* (2.6)
How recently child was measured														
Weight > 6 months ago	11.6	28.2^a^	9.9	19.1^a^	12.7	34.9^a^	9.7	22.1^a^	15.2	27.7	12.4	32.3^a^	9.4	25.0^a^
Height > 6 months ago	18.3	43.0^a^	15.1	32.7^a^	20.6	50.7^a^	14.7	33.3^a^	26.9	32.6	17.7	44.8^a^	17.6	42.9^a^
Mother completed questionnaire	82.6	86.8	80.8	87.6	83.8	86.3	79.3	82.8	85.3	93.7	86.6	91.9	81.2	75.9

EHR = Electronic health record; PR = Has weight classification based on parent-reported data; No PR = Missing weight classification based on parent-reported data.

^a^Significant difference (*P* < .05) between PR and No PR for this demographic subgroup by chi-square test.

### Availability of tools in the home to measure height and weight

Approximately 70% of the households had a scale and 74% a tape measure or yardstick (Table 6). However, only 58% had both of these tools, and 14% had neither. While the availability of these tools in the home did not significantly differ by age group, parents of Black and Latino children were significantly less likely than parents of nonHispanic White and Asian children to report having them.

**Table 6:** **Availability of tools to measure height and weight at home**

		**By child age**		**By child race/ethnicity**			
	**All**	**Ages 3–5 y**	**Ages 6–12 y**	**NonHispanic White**	**Black**	**Latino**	**Asian**
	**(N = 1051)**	**(N = 433)**	**(N = 618)**	**(N = 276)**	**(N = 116)**	**(N = 407)**	**(N = 197)**
	**% (95% CI)**	**% (95% CI)**	**% (95% CI)**	**% (95% CI)**	**% (95% CI)**	**% (95% CI)**	**% (95% CI)**
Scale	69.9 (67.1–72.7)	71.1 (66.8–75.4)	68.2 (65.3–72.6)	76.1 (71.0–81.2)	56.0^a^ (46.9–65.2)	60.9^a^ (56.2–65.7)	82.7 (77.4–88.1)
Tape measure or yardstick	73.8 (71.1–76.5)	76.0 (71.9–80.0)	72.0 (68.5–75.6)	86.6 (82.6–90.6)	65.5^a^ (56.8–74.3)	61.2^a^ (56.5–66.0)	83.7 (78.5–88.9)
Both scale and height measuring tool	57.8 (54.9–60.8)	61.0 (56.4–65.6)	55.7 (51.7–59.6)	71.0 (65.6–76.4)	44.0^a^ (34.8–53.1)	44.5^a^ (39.6–49.3)	73.1 (66.8–79.3)
No tool to measure weight or height	14.3 (12.1–16.4)	13.6 (10.4–16.9)	14.7 (11.9–17.5)	8.3 (5.1–11.6)	22.4^a^ (14.7–30.1)	21.4^a^ (17.4–25.4)	6.5 (3.1–10.1)

CI = Confidence interval.

^a^Significantly differs from nonHispanic Whites (*P* < .05).

## Discussion

In this study of parents’ ability to accurately estimate their children’s height and weight, only 49% of parents who reported their child’s height and 58% who reported their child’s weight in a clinic waiting room survey provided information that matched their child’s height within 1 inch and weight within 2 lbs. Similar to O’Connor and Gugenheim’s clinic based survey [12], Latino children in our clinic-based survey were significantly less likely than nonHispanic White children to have parent-reported height and weight data at this level of accuracy. Children aged 6 to 12 were also significantly less likely than those aged 3 to 5 to have accurately reported weight. Our study, in line with several previous studies [4,5,12,13], found that inaccurate parent-reported weight was more often a result of underestimation than overestimation. However, in contrast to many studies but similar to those of O’Connor and Gugenheim [12] and Shields et al. [13], we found that inaccurate parent-reported height was more likely to result from underestimation than overestimation. Our finding that misclassification of children as obese based on parent-report was associated with underestimation of height is in line with Shields et al. [13].

Approximately 40% of parents did not attempt to estimate their child’s height, about 25% did not attempt to estimate their child’s weight, and fewer than half of the parents provided sufficient information to classify their child’s weight. With the exception of Black children, the percentages of children with and without usable BMI-for-age percentile information from parent reports were similar for overweight/obese and obese classifications based on their EHR. As a consequence, similar to O’Connor and Gugenheim [12], we observed no bias due to missing data for this sample with regard to estimates of overweight/obesity or obesity based on parent-reported data. However, because Black children who were missing parent-reported weight classification data were significantly less likely to be overweight/obese than those without missing data, if the proportion of Black children in the sample had been much larger, there would have been greater potential for bias due to missing data.

As many other studies have found [4,5,7-9,11-15], prevalence of overweight/obesity and obesity among these pre-school and pre-adolescent children based on parent-reported data of height and weight was significantly higher than prevalence based on actual measurements. Similar to the Akinbami and Ogden study that showed larger differences between obesity estimates based on parent-reported versus interviewer-measured height and weight for Black and Mexican-American children than nonHispanic White children [4], we found larger differences for Blacks, Latinos, and Asians than for nonHispanic Whites in prevalence of obesity, but not overweight/obesity, based on parent-reported and EHR data. Despite this overestimation of BMI from height and weight reports, a majority of our parents did not recognize that their child was overweight, consistent with the findings of other studies [21-24]. We found that this misperception was greater for younger than older children, but did not appear to differ by race or ethnicity. We also found that accuracy of parental perception of their child being overweight did not significantly differ for parents who did or did not report usable height and weight data for their children.

Our study adds to knowledge about factors associated with accuracy and availability of parent-reported information about child height and weight. We found that in this clinic-based sample, the source of the parental report (mother vs. father) did not affect accuracy, whether the child was of the same sex or opposite sex of the parent. However, accuracy was significantly associated with length of time since the parent had learned their child’s height and weight, and decrease in accuracy was not linear with time, having the biggest drop off after 7 days. We also found that while parents of Latino, Black, and Asian children were significantly less likely than parents of nonHispanic White children to be able to report their child’s height and weight, accuracy of parent-reported data only differed significantly for Latino children.

We found that only approximately 70% of the parents in our study have sufficient equipment at home to measure weight and height, with significant variation according to race/ethnic groups. This reveals the difficulty in asking parents to obtain and provide accurate data using a scale and tape measure for surveillance, research, and program evaluation if these tools are not provided for this purpose.

A strength of our study is that parent-reported height and weight data and clinic-measured data were obtained on the same day and linked at the individual child level. Because we had EHR data for all children, we were able to compare overweight/obesity and obesity prevalence based on parent-reported data versus measured height and weight data for the whole study population rather than just the subgroup of children who had data from both sources. We examined factors associated with accuracy and unavailability of parent-reported data and showed that length of time since parent last learned the child’s height and weight is the main factor contributing to inaccuracy and lack of reporting. Finally, we described the availability of tools in the home to measure height and weight, showing that less than two-thirds of parents of Black and Latino children reported having a scale at home and approximately one in five a scale or tape measure/yardstick. The large percentage of families lacking a scale at home suggests that researchers and pediatricians should not assume that most parents with overweight and obese children currently have sufficient tools to monitor their child’s weight at home.

The main limitation of this study is the large percentage of children whose parents did not provide usable height and weight data. We used the situation that approximately half of the children did not have a usable BMI-for-age percentile to classify them as overweight/obese or obese as an opportunity to examine the issue of potential bias introduced by missing data. However, missing data affected our ability to assess accuracy of parent-report compared to the EHR, especially for the Black and Asian subgroups. The small size of our Latino, Black, and Asian subgroups with parent-reported data also limited our ability to assess differences in accuracy by child sex and age within race/ethnic group. Because our results are based on samples of patients seen in three pediatric clinics of a large Northern California health plan, the racial and ethnic composition of the sample may not be generalizable to other populations. Finally, while we used the EHR as our “gold standard” for height and weight, we cannot be sure that all measurements were taken and recorded accurately by the clinic medical assistants.

## Conclusions

In this study, use of parent-reported height and weight data overestimated the prevalence of childhood overweight/obesity and obesity compared to clinic-measured data. Due to cost and logistical constraints it may not be possible to base estimates of overweight and obesity prevalence for national, state, or local initiatives on recently measured height and weight data, but our results add to a growing number of studies that recommend that parent-reported data not be used to estimate prevalence of overweight/obesity and obesity among pre-school and elementary school aged children.

## Additional file

Additional file 1:
**Survey Questionnaire.**
