# Supplementary material for: Accuracy of parent-reported information for estimating prevalence of overweight and obesity in a race-ethnically diverse pediatric clinic population aged 3 to 12
Source: BMC Pediatr. 2015 Feb 12;15:5. doi: 10.1186/s12887-015-0320-0 (PMC4341231; doi:10.1186/s12887-015-0320-0)
Supplement: Additional file 1: — Survey Questionnaire. [file 12887_2015_320_MOESM1_ESM.pdf]

KAISER PERMANENTE®

PARENT SURVEY ABOUT CHILD'S HEIGHT AND WEIGHT

Kaiser Permanente's Division of Research is doing a study to learn how accurate parents are when they are asked to report their child's height and weight in surveys and to medical staff during phone consults.

We hope you will agree to fill out this questionnaire. When you are done, you will receive a small gift to thank you for your help. We will link your answers about your child's height and weight to the height and weight measured by the medical assistant today. All of this information will be kept confidential.

You do not need to answer these questions if you do not want to. If you decide not to, neither your child's medical care nor Health Plan benefits will be affected.

1) How old is your child? _______ **Child's MRN**: ___________________

2) Is your child a: ☐ Boy ☐ Girl

3) How tall is your child (without shoes)?
____ feet ___ inches **OR** ____ meters ___ cm **OR** ☐ Don't know

4) How much does your child weigh (without shoes)?
____ pounds **OR** _____ kilograms ___ grams **OR** ☐ Don't Know

5) How confident are you that your answers about your child's height and weight are correct?
a. Height: ☐ Very confident ☐ Fairly confident ☐ Not confident
b. Weight: ☐ Very confident ☐ Fairly confident ☐ Not confident

6) When was the last time you found out your child's height?
☐ In the past 7 days ☐ In the past month ☐ In the past 6 months ☐ More than 6 months ago

7) Do you think your child has grown taller since the last time he/she was measured?
☐ No taller ☐ A little bit taller ☐ A lot taller

8) When was the last time you found out your child's weight?
☐ In the past 7 days ☐ In the past month ☐ In the past 6 months ☐ More than 6 months ago

9) Do you think your child is:
☐ Underweight ☐ About the right weight ☐ Overweight ☐ Very Overweight

10) Do you have a scale at your home that could be used to weigh your child if you were asked to do so?
☐ Yes ☐ No

11) Do you have a tape measure or yardstick at your home that could be used to measure your child's height if you were asked to do so?
☐ Yes ☐ No

12) What size jeans or uniform/dress pants does your child currently wear? *(Circle your answer)*
2T 3T 4T 5T 4 5 6 7 8 9 10 12 14 16 18 20
Is this size: ☐ Regular ☐ Slim ☐ Husky

13) Who completed this questionnaire?
☐ Mother ☐ Father ☐ Other: _________________________________
